# Cobalt Oxide and Cobalt‐Graphitic Carbon Core–Shell Based Catalysts with Remarkably High Oxygen Reduction Reaction Activity

**DOI:** 10.1002/advs.201600060

**Published:** 2016-04-23

**Authors:** Jie Yu, Gao Chen, Jaka Sunarso, Yinlong Zhu, Ran Ran, Zhonghua Zhu, Wei Zhou, Zongping Shao

**Affiliations:** ^1^ Jiangsu National Synergetic Innovation Center for Advanced Materials (SICAM)State Key Laboratory of Materials‐Oriented Chemical EngineeringCollege of Chemistry and Chemical EngineeringNanjing Tech UniversityNo. 5 Xin Mofan RoadNanjing210009P. R. China; ^2^ Department of Chemical Engineering, Curtin UniversityPerthWestern Australia6845Australia; ^3^ School of Chemical EngineeringThe University of Queensland, St. LuciaQueensland4072Australia

**Keywords:** cobalt, core–shell structure, ethylenediaminetetraacetic acid, non‐precious catalyst, oxygen reduction reaction

## Abstract

**The vital role of ethylenediaminetetraacetic acid** on the structure and the oxygen reduction reaction activity of the non‐precious‐metal‐based pyrolyzed catalyst is reported and elaborated. The resultant catalyst can overtake the performance of commercial Pt/C catalyst in an alkaline medium.

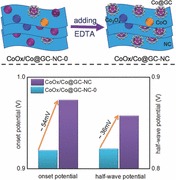

Polymer electrolyte membrane fuel cell (PEMFC) offers much higher fuel efficiency and lower point‐of‐use emission over the combustion engine.^1^ These advantages, in addition to longer operating time and fast refueling, render it more competitive than the conventional battery devices. PEMFC has indeed drawn a growing market share in light duty vehicle, bus and material handling vehicle propulsion as well as in back‐up and remote power systems.^1^ The efficiency and performance of the widely used low temperature version of this device, however, is limited by the oxygen (O_2_) reduction reaction (ORR) at or near ambient temperature on its cathode side.^2^ To overcome the sluggish nature of the ORR kinetics, the presence of an efficient electrocatalyst is required.^3^ Pt has, up to now, dominated the cell cost due to its status as the ORR catalyst benchmark despite its high cost which prohibits large scale deployment of PEMFC technology.^4^ As a noble metal, its cost is independent of the production quantity scale which hinders the economic pricing of PEMFC stack.[[qv: 5a]]

The replacement of Pt and/or Pt alloys by cheaper metal‐free elements or non‐precious transition metals (i.e., Fe, Co, etc.) currently lies at the forefront of the ORR catalyst research and development.^5^ One of the most promising candidates is metal and nitrogen co‐doped carbon (M, N—C; M = metal)‐based electrocatalysts given their low cost, excellent catalytic activity, and high durability, which was synthesized via high‐temperature pyrolysis of M, N, and C containing precursors such as porphyrin, aniline, chitosan, and/or metal‐organic frameworks.[[qv: 5e–m]] A new concept of M, N—C catalyst featuring metal‐based nanoparticles (CoO*_x_*, FeO*_x_*_,_ and FeC*_x_*) encapsulated in nanostructured graphitic carbon was recently reported; the performances of which are comparable or even exceed that of Pt/C either in terms of a more positive onset potential, a more positive half‐wave potential, or a higher limiting current density.[[qv: 5h–m]] At present, only a few materials can surpass Pt/C performance simultaneously on these three ORR parameters. In these materials, the uniform graphitic layers generally wrap the metal‐based nanoparticles (NPs), forming core–shell structure which prevents direct exposure of NPs to O_2_ and/or electrolyte and therefore effectively enhancing the chemical stability of the active components in harsh environments. Additionally, those NPs appear to activate the catalytic activity of the encapsulating graphitic layers, conceptually via electron density modification.[[qv: 5h,l]] Notably, the nature of the interfacial contact and dispersion between the metal cores and the carbon shells plays a key role toward optimizing the ORR activity. Nitrogen‐doped carbon also favors enhanced ORR activity which was attributed to the activity of the pyridinic nitrogen sites by most studies.^5^, ^6^ Distinct N active sites were reported to have distinct role where typically pyridinic N contributes to improved onset potential whereas graphitic N facilitates enhanced current density.^7^ Other N sites, such as pyrrolic N and oxidized N, may also exist, but they are not reported to be catalytically active. Furthermore, high specific surface area is considered important to attain more active ORR sites.^8^

Here, we report that a uniform distribution of cobalt oxide nanoparticles and cobalt metal nanoparticles–graphitic carbons (core–shell structure) sandwiched between nitrogen‐doped carbon sheets (designated as CoO*_x_*/Co@GC‐NC) can be synthesized via a facile one‐pot pyrolysis route using cobalt (II) nitrate hexahydrate, d‐glucosamine hydrochloride (GAH), ethylenediaminetetraacetic acid (EDTA) and melamine as the precursors. The indispensable role of EDTA toward achieving such a unique structure is substantiated by characterizing the resultant material from identical synthesis procedure, but without EDTA addition, which we designate as CoO*_x_*/Co@GC‐NC‐0. We elaborate below that EDTA contributes toward improved cobalt metal encapsulation by graphitic carbon, increased specific surface area in addition to increased nitrogen active sites, i.e., pyridinic N. The unique structure of this material, possessing the synergistic effects, gives rise to excellent ORR activity, long‐term stability, and resistance to methanol. These merits render a better performance of CoO*_x_*/Co@GC‐NC in an alkaline medium compared with Pt/C electrocatalyst.

CoO*_x_*/Co@GC‐NC was prepared via a soft‐template method using a single‐step thermal condensation‐annealing of cobalt nitrate, GAH, melamine, and EDTA mixture at 800 °C in an inert N_2_ atmosphere. GAH and EDTA act as the carbon and nitrogen sources whereas cobalt (II) nitrate hexahydrate serves as the cobalt oxide and cobalt metal sources. Melamine, on the other hand, acts as the soft template during the *in situ* synthesis of N‐doped graphitic carbon.^9^ The underlying concept of the pyrolysis route we used here has been described elsewhere.^9^ The novelty of this work lies in the preparation and ORR characterization of the novel cobalt‐based analogue which relies on the addition of EDTA to achieve optimized ORR performance. A schematic diagram describing the catalysts syntheses (CoO*_x_*/Co@GC‐NC and CoO*_x_*/Co@GC‐NC‐0) is shown as **Scheme** **1**. In retrospect, during the thermal condensation, melamine was polymerized into graphitic carbon nitride layers. Concurrently, cobalt nitrate was decomposed into cobalt oxide and metallic cobalt nanoparticle while GAH and EDTA were simultaneously decomposed into nitrogen‐doped carbon skeleton wrapping these nanoparticles; all of which were distributed within the layers of the carbon nitride.^9^

**Scheme 1:**
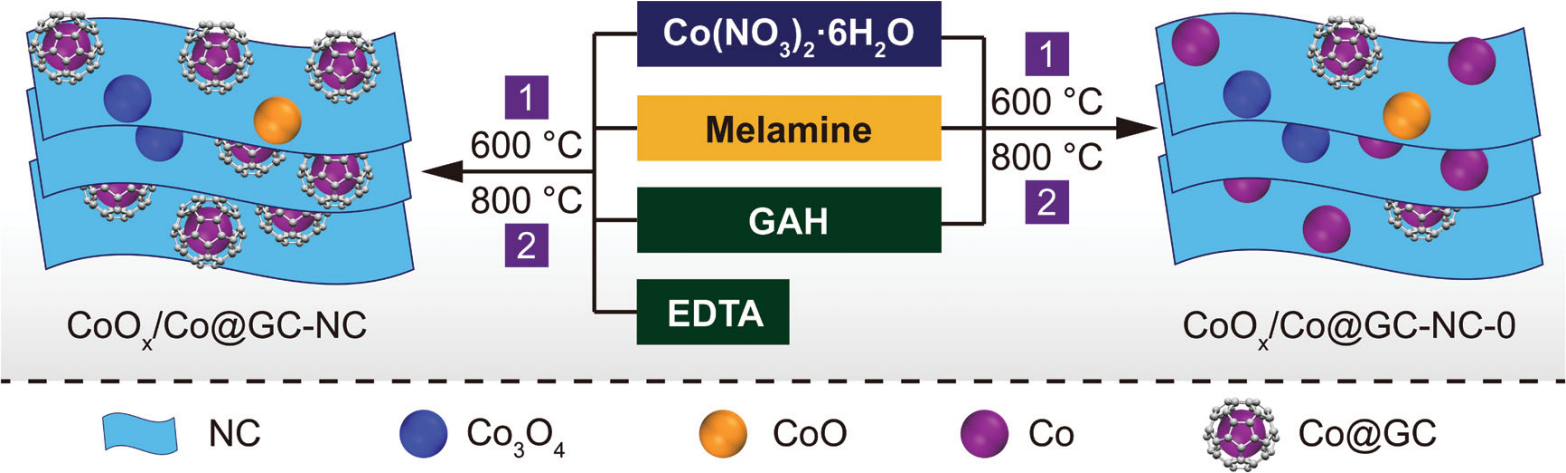
Pictorial diagram of the syntheses of CoO*_x_*/Co@GC‐NC and CoO*_x_*/Co@GC‐NC‐0.

Scanning electron microscope (SEM) and transmission electron microscope (TEM) images (**Figure** **1**a,b) indicate that Co‐based nanoparticles with a diameter of 5–20 nm are uniformly dispersed and anchored on N‐doped graphitic carbon matrix. The close interfacial contact developed between the Co‐based NPs and the carbon sheets would favor enhanced charge transfer. Distinct lattice fringe spaces of 2.12 and 4.67 Å, corresponding to the (200) plane of CoO and the (111) plane of Co_3_O_4_, respectively, appeared in the following high resolution TEM (HR‐TEM) images (Figure 1c). Figure 1c additionally shows another sheet‐like fringe with an interplanar distance of 2.04 Å which represents the (111) plane of Co^0^ (i.e., Co metal). The complete encapsulation of such metal NPs by graphene sheet‐like carbon layers rationalizes their passivity toward oxidation as we will discuss below (Figure 1d). Figure 1e further focuses on the carbon layers in the vicinity of NPs which highlights the highly carbonized nature of the sample. Elemental mapping of the sample verifies the uniform distribution of Co, C, N, and O on their expected sites (Figure S2, Supporting Information).

**Figure 1:**
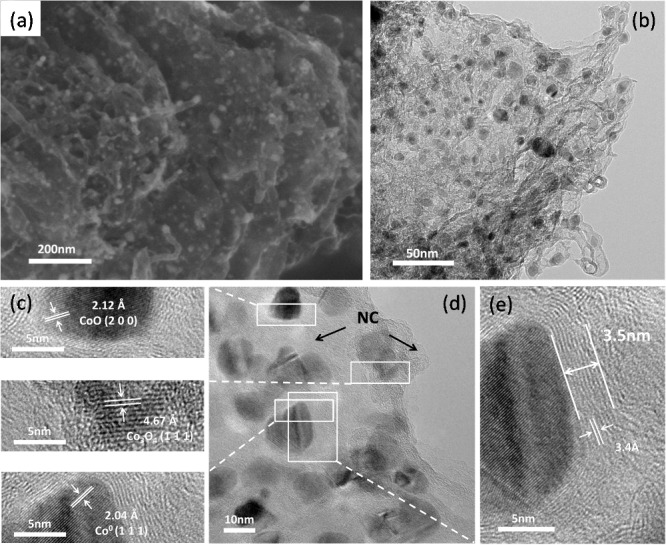
a) Scanning electron microscope image; b) transmission electron microscope image; and c–e) high resolution transmission electron microscope images of CoO*_x_*/Co@GC‐NC.

To identify the constituent phases of CoO*_x_*/Co@GC‐NC and CoO*_x_*/Co@GC‐NC‐0, powder X‐ray diffractions (XRD) were performed as depicted in **Figure** **2**a. The major peaks on both patterns can be assigned to Co (i.e., 44.16° and 51.47° peaks), CoO (i.e., 42.39° and 61.44° peaks), and Co_3_O_4_ (i.e., 18.83°, 31.24°, 36.79°, 55.56°, 59.31°, and 65.26° peaks). These peaks appear more intense for CoO*_x_*/Co@GC‐NC (compared to CoO*_x_*/Co@GC‐NC‐0) which indicates higher content of these phases. This is consistent with the higher background noise for the former pattern due to the significant Co fluorescence. Disordered (amorphous) carbon also exists in both samples as implied by the broad hump featured on both patterns.^10^ Another visible difference between the two patterns is a peak at 26.1° attributed to the formation of graphitic carbon, indicating good carbonization and is in accordance with the HR‐TEM images (Figure 1e). The presence of graphitic carbon layers is essential toward enhanced electrical conductivity.

**Figure 2:**
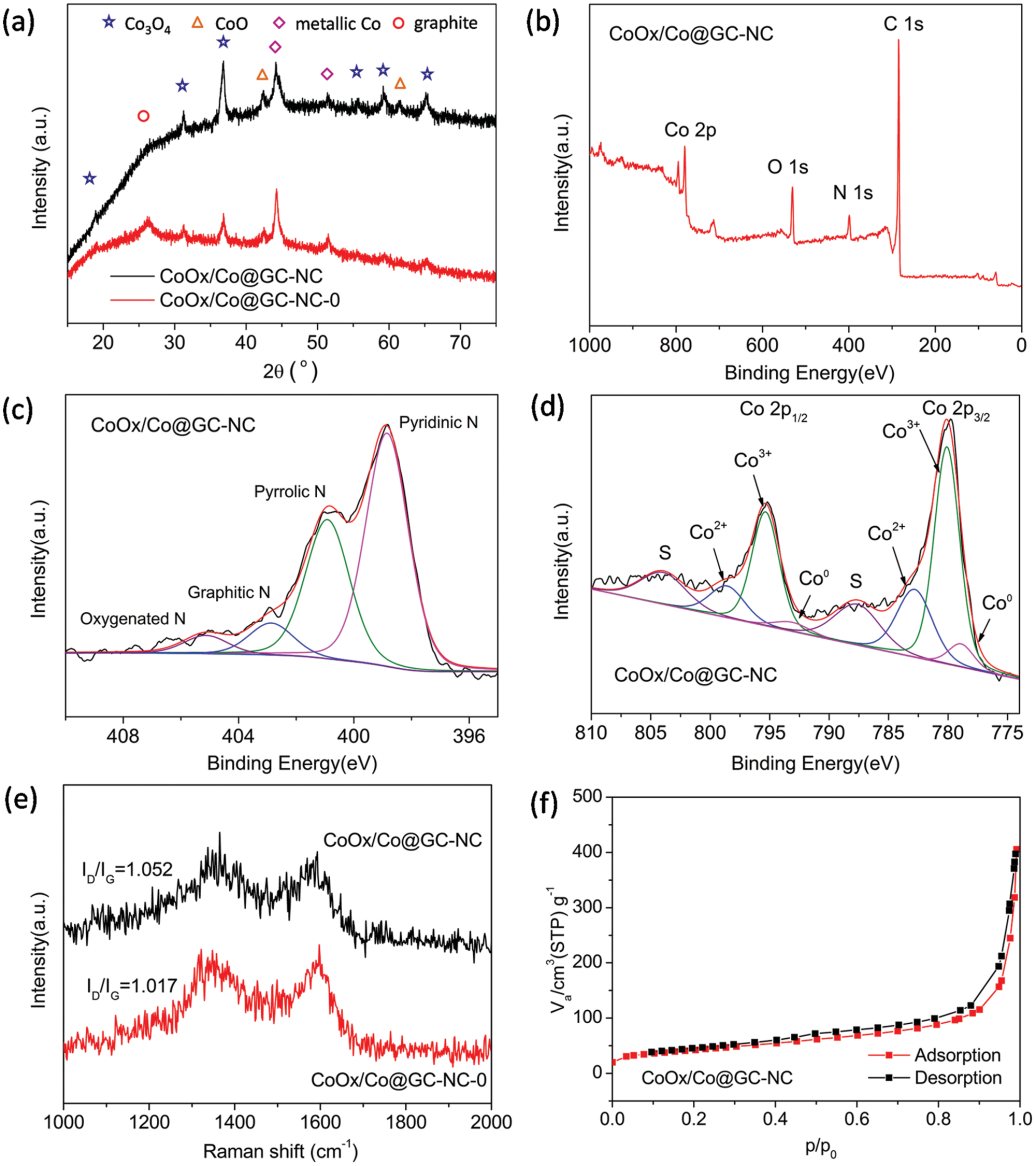
a) Powder X‐ray diffraction patterns of CoO*_x_*/Co@GC‐NC and CoO*_x_*/Co@GC‐NC‐0. b) X‐ray photoelectron spectroscopy wide‐scan spectrum of CoO*_x_*/Co@GC‐NC. c,d) High‐resolution N 1s and Co 2p XPS spectra of CoO*_x_*/Co@GC‐NC. e) Raman spectra of CoO*_x_*/Co@GC‐NC and CoO*_x_*/Co@GC‐NC‐0. f) N_2_ adsorption–desorption isotherms of CoO*_x_*/Co@GC‐NC.

Using X‐ray photoelectron spectroscopy (XPS), the composition of the most ORR active species, i.e., nitrogen and cobalt species can be determined. The wide‐scan spectrum (Figure 2b) corroborates the existence of C, N, Co, and O elements. N 1s spectra follow next **(**Figure 2c), which can be deconvoluted into pyridinic N (398.8 eV), pyrrolic N (400.9 eV), graphitic N (402.9 eV), and oxidized N (405.1 eV) with the relative atom ratios of 54.5%, 33.5%, 7.7%, and 4.3%, respectively. Co 2p spectra (Figure 2d) reveal two distinct energy bands profile, i.e., a high energy band at 795.5 eV and a low energy band at 780.1 eV, which can be attributed to Co 2p 3/2 and Co 2p 1/2, respectively. These doublets can be deconvoluted into two prominent peaks, i.e., around 780.1 and 795.4 eV for Co^3+^ and around 782.8 and 798.6 eV for Co^2+^. Cobalt metal presence, which is previously detected in powder XRD and TEM results, nonetheless, appeared to be absent in Co 2p spectra (Figure 2d). Its detection here is difficult due to its very low content in addition to its close vicinity to the Co^3+^ profile. As we will discuss later, Co° clearly exists in detectable amount in Co 2p spectra of CoO*_x_*/Co@GC‐NC‐0. The two small visible peaks at 787.8 and 803.8 eV, marked with S, represent Co^2+^ shake‐up satellite peaks.[[qv: 5h]]

Raman spectra provide further evidence of the partial graphitization of carbon shell structure (Figure 2e) which displays two characteristic peaks centered at approximately 1350 cm^−1^ (D band) and 1590 cm^−1^ (G band). The G‐band originates from the graphitic structure whereas the D‐band comes from the defects on the disordered carbon.^5^, ^11^ The calculated intensity ratios of D/G band (*I*_D_/*I*_G_) of 1.052 for CoO*_x_*/Co@GC‐NC is indicative of a relatively high amount of structural defects accompanied by approximately similar content of ordered graphitic structure. Higher defects content has often been correlated with the ORR activity enhancement.[[qv: 9b]]

Turning to the N_2_ sorption isotherms (Figure 2f) and the respective pore size distribution (Figure S3, Supporting Information – obtained using Barrett–Joyner–Halenda model), it is apparent that CoO*_x_*/Co@GC‐NC exhibits an isotherm characteristic of mesoporous materials (type IV isotherm) which features hysteresis between its adsorption and desorption profile.This material shows a Brunauer–Emmett–Teller specific surface area of 148.03 m^2^ g^−1^. CoO*_x_*/Co@GC‐NC‐0 has a lower specific surface area of 119.9 m^2^ g^−1^ (Table S1, Supporting Information). In view of the availability of more active sites, higher surface area is of interest.^8^ Moreover, the presence of meso and/or macroporosity have been demonstrated to promote the transport of ORR‐related species (O_2_, H^+^, OH^−^, and H_2_O).[[qv: 8c]],^12^

To evaluate the catalytic activity, cyclic voltammetry in O_2_ versus Ar‐saturated were initially performed in 0.1 m KOH solution (**Figure** **3**a). Every potential scale in the electrochemical data in this work is referenced against reversible hydrogen electrode (details in the Experimental section and Figure S1 in the Supporting Information). The tested catalysts exhibit cathodic peak currents only under O_2_‐saturated condition; consistent with its ORR origin. Larger limiting current density and ≈10 mV more positive onset potential for CoO*_x_*/Co@GC‐NC relative to CoO*_x_*/Co@GC‐NC‐0 reflect the higher ORR activity for the former catalyst. The larger capacitive current (i.e., non‐zero current contribution observed in the absence of ORR during Ar‐saturated scan) for the former catalyst relative to the latter one is additionally consistent with the larger surface area for the former catalyst.^5^

**Figure 3:**
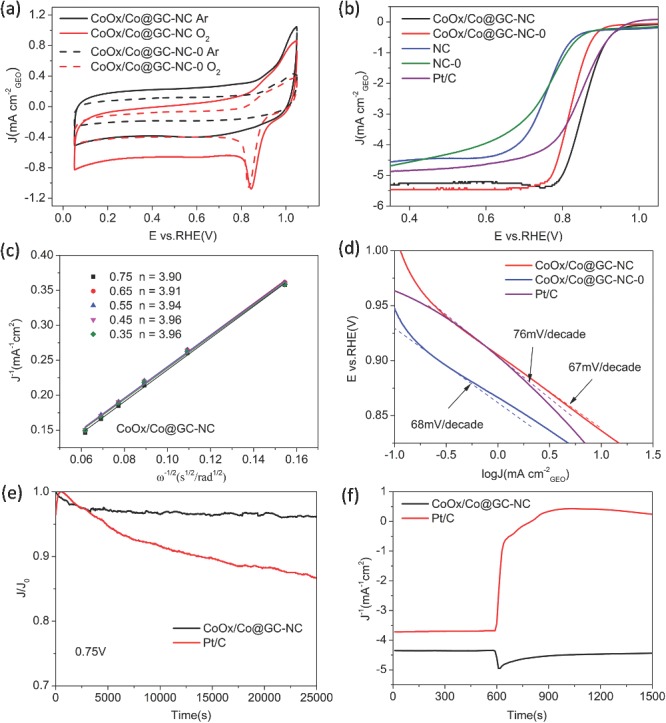
a) Cyclic voltammetry profiles of CoO*_x_*/Co@GC‐NC and CoO*_x_*/Co@GC‐NC‐0 in an O_2_ and Ar‐saturated 0.1 m KOH solution at a scan rate of 10 mV s^−1^. b) Linear sweep voltammetry profiles of CoO*_x_*/Co@GC‐NC and CoO*_x_*/Co@GC‐NC‐0 (two catalysts) and NC, NC‐0, and commercial 20 wt% Pt/C (three control materials) obtained using rotating disk electrode at 1600 rpm in an O_2_‐saturated 0.1 m KOH solution at a scan rate of 5 mV s^−1^. c) The respective Koutecky–Levich plots at different potentials for CoO*_x_*/Co@GC‐NC derived from Figure S4a (Supporting Information). d) Tafel plots of CoO*_x_*/Co@GC‐NC, CoO*_x_*/Co@GC‐NC‐0. and commercial 20 wt% Pt/C. e) Chronoamperometric response of CoO*_x_*/Co@GC‐NC and commercial 20 wt% Pt/C at 0.75 V obtained using rotating disk electrode at 1600 rpm in an O_2_‐saturated 0.1 m KOH solution. f) Chronoamperometric response of CoO*_x_*/Co@GC‐NC and commercial 20 wt% Pt/C at 0.75 V in an O_2_‐saturated 0.1 m KOH solution without methanol (0−600 s) and with the addition of 3 m methanol (600−1500 s) obtained using rotating disk electrode at 1600 rpm.

We then assess the diffusion‐limited current density and the onset potential of ORR using rotating disk electrode (RDE). Linear sweep voltammetry (LSV) profiles for two different catalysts and three control materials, i.e., CoO*_x_*/Co@GC‐NC, CoO*_x_*/Co@GC‐NC‐0, nitrogen‐doped carbon sheets (NC), nitrogen‐doped carbon sheets obtained without EDTA addition (NC‐0) and Pt/C are depicted in Figure 3b. As a note, NC and NC‐0 are the materials obtained from identical synthesis to CoO*_x_*/Co@GC‐NC, except with the absence of cobalt (II) nitrate hexahydrate. The ORR current profiles for NC and NC‐0 are quite identical. These nitrogen‐doped carbons exhibit ≈0.15 V more negative onset potential than the other metal containing catalysts. They also show similar values of diffusion‐limited current densities, i.e., ≈4.6 mA cm^−2^ which signifies an apparently less than but close to four‐electron ORR process; provided that the current density value for an apparent four‐electron process is ≈6 mA cm^−2^ at 1600 rpm according to Levich equation.^13^ This observation mirrors recent reports on nitrogen‐doped graphitic carbon.[[qv: 6b]],^14^ CoO*_x_*/Co@GC‐NC‐0 shows a higher ORR activity over these carbons with an onset potential of 0.920 V (at −0.15 mA cm^−2^) and a half‐wave potential of 0.822 V. The most active catalyst is CoO*_x_*/Co@GC‐NC, which shows an onset potential of 0.974 V (at −0.15 mA cm^−2^) and a half‐wave potential of 0.858 V. This comes somewhat surprisingly because ≈0.054 V higher ORR activity can be obtained via EDTA addition. The ORR performance of CoO*_x_*/Co@GC‐NC is comparable to the best one reported and exceeds most metal and nitrogen co‐doped carbon materials in an alkaline medium (refer to Table S3 in the Supporting Information for comparison).[[qv: 5h–j]],[[qv: 8b]],^15^ More significantly, relative to Pt/C, CoO*_x_*/Co@GC‐NC clearly demonstrates higher diffusion‐limited current density, more positive half‐wave potential in addition to identical onset potential; highlighting its superior performance against the benchmark ORR catalyst. At present, only a handful amount of materials can overtake Pt/C performance, most of which are derived from precious metal components such as Au and Pd.^16^

The Koutecky–Levich experiments of CoO*_x_*/Co@GC‐NC involves getting LSVs at different rotation rate (Figure S4a, Supporting Information). When the inverse of the current density is plotted against the inverse of the root square of the rotation rate (Figure 3c), analogous slopes were obtained from several data taken at different potential, signifying the first‐order reaction kinetics of the ORR process.[[qv: 15c]] The calculated slopes show negligible variation, i.e., 3.90–3.96, again in agreement with its current density magnitude and four‐electron process. We also cross‐check the hydrogen peroxide formation on the two catalysts by rotating ring‐disk electrode measurements (Figure S4d, Supporting Information) which despite the observed minor disparity, detect only a minor fraction of peroxide species.

Kinetic data in terms of Tafel plots is displayed next (Figure 3d). In the perspective of achieving high current at low overpotential, lower slope becomes an indicator of better catalytic performance.^17^ A Tafel slope of 67 mV per decade was measured for CoO*_x_*/Co@GC‐NC which is quite identical to that for CoO*_x_*/Co@GC‐NC‐0 (68 mV per decade) and compares favorably against Pt/C (76 mV per decade).

Durability is another key parameter for a practical catalyst. We subjected the best catalyst and Pt/C to 25 000 s (≈7 h)‐chronoamperometry test using RDE in an O_2_‐saturated 0.1 m KOH solution at a rotation rate of 1600 rpm by fixing the potential at 0.75 V and monitor its current (Figure 3e). Unlike Pt/C, which retains only 86% of its initial current at 25 000 s, CoO*_x_*/Co@GC‐NC shows a much lower current fading rate with 94% retention. In particular, the current profile for the latter catalyst appears to stabilize following the initial degradation during the first 4000 s while the current for Pt/C continuously degrades over the test duration. The enhanced corrosion resistance is likely contributed by the graphitic carbon layer as reported elsewhere.[[qv: 5j]],[[qv: 8c]]

One of the other main advantages that Pt/C provides is its applicability to be used as a cathode on methanol‐fueled cell in addition to H_2_‐fueled one. Pt/C is however prone to poisoning by methanol which may cross over from anode to cathode side. CoO*_x_*/Co@GC‐NC does not have such drawback **(**Figure 3f). Chronoamperometry tests confirms the major degradation of the ORR current of Pt/C, once 3 m methanol solution is added into 0.1 m KOH electrolyte at 600 s (≈10 min), which is not observed for the most active catalyst case. The tendency to participate in methanol oxidation is related with the OH formation property of the catalyst.^18^ Pt is much more active in this regard whereas carbon‐based materials are typically not.[[qv: 5g,h]]

The fact that CoO*_x_*/Co@GC‐NC is much more electrochemically active compared to CoO*_x_*/Co@GC‐NC‐0 raises questions on the role of EDTA and the contributing mechanisms. SEM images (Figure 1a; Figure S5, Supporting Information, for CoO*_x_*/Co@GC‐NC‐0) comparison does not show major difference in terms of morphology. Powder XRD patterns comparison, as already discussed above, indeed provides insight into an enhanced formation of Co‐based NPs (Figure 2a). N_2_ sorption isotherms reveal that, relative to CoO*_x_*/Co@GC‐NC‐0, CoO*_x_*/Co@GC‐NC shows enhanced pores formation, i.e., 23% higher specific surface area and 46% higher pore volume (see Table S1 in the Supporting Information for the summary of values and compare Figure 2f with Figure S6, Supporting Information for the control materials which include CoO*_x_*/Co@GC‐NC‐0, NC, and NC‐0). Higher surface area obtained through EDTA addition allows higher exposure to more active sites, therefore effectively contributing toward the ORR enhancement. Raman spectra (Figure 2e) further indicate slightly higher *I*_D_/*I*_G_ value for CoO*_x_*/Co@GC‐NC compared to CoO*_x_*/Co@GC‐NC‐0. To this end, the former material has more defective sites in its carbon structure which serves as another attribute that adds to the higher ORR activity.[[qv: 9b]] A more detailed probing into Co 2p XPS spectra of CoO*_x_*/Co@GC‐NC in comparison to CoO*_x_*/Co@GC‐NC‐0 (Figure 2d; Figure S7b, Supporting Information) highlights a significant formation of Co metal in the latter material which appears to be absent (or below the detection limit) in the former material. This is in contrast to the powder XRD and TEM analyses corroborating Co metal presence. The most plausible explanation for this contradiction is that in CoO*_x_*/Co@GC‐NC‐0, a great proportion of Co metals are not completely encapsulated by the graphitic carbon whereas practically all Co metals in CoO*_x_*/Co@GC‐NC are enclosed within the graphitic shell. In another word, EDTA addition promotes the encapsulation process of Co by graphitic carbon. We attribute this to the chelation of Co (III) by EDTA in aqueous solution forming essentially six‐coordination complexes of Co center with two nitrogens and four oxygens from EDTA which have unpaired electrons.^19^ N 1s XPS spectra provide an additional hint into the possibility of ORR activity contribution from nitrogen‐doped carbon as reported in several works.^5^, ^6^, ^7^ Comparison between the spectra of CoO*_x_*/Co@GC‐NC and CoO*_x_*/Co@GC‐NC‐0 (Figure 2c; Figure S7c, Supporting Information; the results of which are summarized in Table S2, Supporting Information) establishes the dominant presence of pyridinic nitrogen in the most active catalyst. This result is in accord with several reports that attribute the ORR activity of N‐doped carbons to pyridinic N content and show the dominant role of this N to enable carbon atoms next to it become the active sites for ORR, because the carbon atom next to the pyridinic N has the highest probability to adsorb the oxygen molecule followed by the protonation of the adsorbed O_2_.[[qv: 6b]],^14^ EDTA addition thus also enhanced the pyridinic N content which contributes to the ORR activity enhancement.

Additional insights pertaining to the nitrogen content and the effect of the precursor choice into the distribution of distinct N species can be obtained by looking at Figure S8 and Table S2 (Supporting Information). In addition to CoO*_x_*/Co@GC‐NC and CoO*_x_*/Co@GC‐NC‐0, three other control materials, i.e., the resultant material from identical synthesis procedure but obtained by replacing EDTA with citric acid (CA) (designated as CoO*_x_*/Co@GC‐NC‐1), the pyrolysis product from a mixture of cobalt (II) nitrate hexahydrate and EDTA (Co‐EDTA), and the pyrolysis product from a mixture of cobalt (II) nitrate hexahydrate and citric acid (Co‐CA). The XPS and elemental analysis display analogous trend despite their values disparity which reflects their distinct sample detection natures, i.e., one is surface and another is bulk analysis. The equally high N content of Co‐EDTA and Co‐CA, in fact, the highest among the tested materials (Figure S8 and Table S2, Supporting Information), comes from the necessity to add a relatively high amount of ammonia to obtain homogeneous solution of cobalt metal nitrate with the chelating agent (EDTA or CA). As expected, in terms of N content, CoO*_x_*/Co@GC‐NC has the highest content followed by CoO*_x_*/Co@GC‐NC‐1 and CoO*_x_*/Co@GC‐NC‐0, noting the lack of nitrogen elements in CA. In view of maximizing the relative amount of pyridinic N, adding chelating agent appears beneficial (Table S2, Supporting Information). Still in this context, EDTA works better than CA.

Returning to the ORR evaluation, LSVs were performed on CoO*_x_*/Co@GC‐NC‐1, Co‐EDTA, and Co‐CA. Relatively poor performance was obtained for Co‐EDTA and Co‐CA (Figure S9, Supporting Information) plausibly due to their very low specific surface areas (see Table S1 and the respective N_2_ sorption isotherms in Figure S10 in the Supporting Information). High surface area Co‐EDTA and Co‐CA, obviously, cannot be achieved via pyrolysis route. The absence of Co metal and oxide components on the powder XRD patterns of Co‐EDTA and Co‐CA is counterintuitive (Figure S11, Supporting Information) as the same amount of cobalt precursors (relative to synthesis of CoO*_x_*/Co@GC‐NC) was used. XPS supports this observation, i.e., by detecting only a very low amount of Co atoms of 0.56 at% in Co‐EDTA and 0.41 at% in Co‐CA (Figure S12, Supporting Information). We speculate that the pyrolysis temperature of 800 °C (which we fix at this value to be consistent with the other samples syntheses) may not be sufficiently high to crystallize the cobalt‐based particles.

To further determine the effect of different Co components on the ORR, we subjected CoO*_x_*/Co@GC‐NC to etching and oxidation. By exposing CoO*_x_*/Co@GC‐NC to 3 m HCl for 72 h at room temperature, cobalt oxide components can be effectively removed from the original matrix; the resultant sample of which hereby designated as Co@GC‐NC. This is evident in the powder XRD pattern (**Figure** **4**a) for Co@GC‐NC which retains peaks from Co metal only. What makes Co metals components relatively unscathed following such harsh treatment is the robust nature of its encapsulation by graphitic carbon. Co@GC‐NC indeed shows very high ORR activity through an apparent four‐electron process which however is still lower than the original CoO*_x_*/Co@GC‐NC catalyst given its more negative onset potential (Figure 4b). This is somewhat surprising and leads us to think that the simultaneous coexistence of cobalt metal and cobalt oxides induces synergy effect in the creation of active sites for ORR as also reported elsewhere.[[qv: 5h]],^20^ We were not successful in our next attempt to remove Co metal from Co@GC‐NC by subjecting it to 12 m HCl for 192 h at 60 °C (designated as Co@GC‐NC‐12 m HCl‐192 h). The subsequent powder XRD (Figure S13, Supporting Information) indicates the retaining of Co metal in the matrix of Co@GC‐NC‐12 m HCl‐192 h. It is worth noting that Co metal‐graphitic carbon core–shell actually provides an ideal configuration where Co core is not exposed to oxygen or electrolyte (prevents its attack) yet is able to modify the electron density of the surrounding graphene layers to activate their ORR activity.[[qv: 5h,l]] The importance of graphitic carbon shells has been elaborated in a recent study involving cobalt components within a highly ordered porous carbon matrix.[[qv: 5h]] Moreover, the intimate contact between Co core and carbon shell hinders the possible agglomeration of metal NPs at high temperature and also facilitate the electron transport from nitrogen‐doped carbon sheets to Co cores, leading to a synergistic effect toward oxygen reduction reaction.[[qv: 5m]],[[qv: 8c]],^21^ The LSV of Co@GC‐NC‐12 m HCl‐192 h again resembles that of Co@GC‐NC (Figure 4b). Aiming to test the possible oxidation of Co core to cobalt oxide, our last resort was the oxidation of CoO*_x_*/Co@GC‐NC in air at 250 °C for 192 h, resulting in the sample we designate as CoO*_x_*/Co@GC‐NC‐250 °C‐192 h. Despite the partial oxidation possibility, Co still predominantly appears in the powder XRD pattern of CoO*_x_*/Co@GC‐NC‐250 °C‐192 h (Figure S13, Supporting Information). Nonetheless, its LSV profile indeed shows substantial reduction in the ORR activity, against the original material; which approaches the profiles of the acid leached catalysts (Figure 4b). This, we speculate, originates from the oxidation of CoO and some Co metal cores.

**Figure 4:**
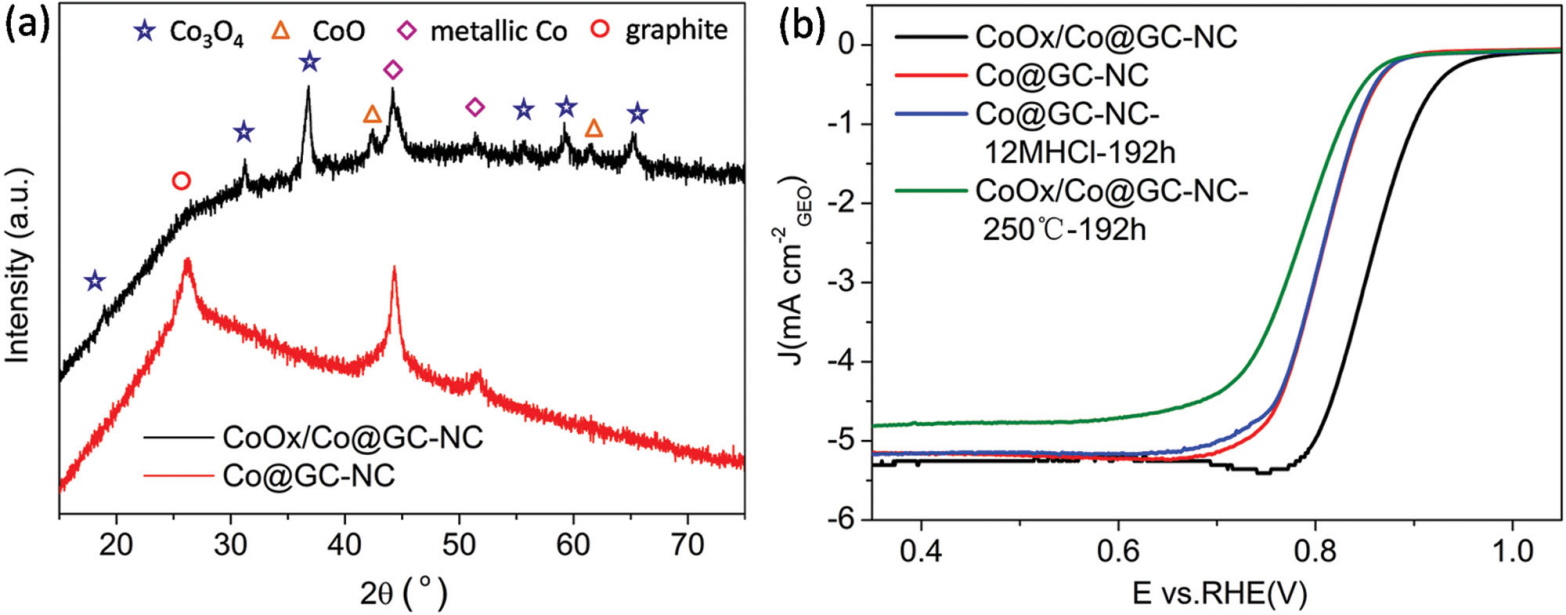
a) Powder X‐ray diffraction patterns of CoO*_x_*/Co@GC‐NC and Co@GC‐NC. b) Linear sweep voltammetry profiles of CoO*_x_*/Co@GC‐NC, Co@GC‐NC, Co@GC‐NC‐12 m HCl‐192 h, and CoO*_x_*/Co@GC‐NC‐250 °C‐192 h obtained using rotating disk electrode at 1600 rpm at different rotation rate in an O_2_‐saturated 0.1 m KOH solution at a scan rate of 5 mV s^−1^.

CoO*_x_*/Co@GC‐NC demonstrated very high ORR performance, only shown by a handful of catalysts at present. Different characterization results presented here demonstrate the accumulative effects EDTA provide to enable such excellent performance, i.e., higher surface area (more active sites), more defective sites on the carbon structure, more complete encapsulation of Co metal core by graphitic carbon shell, and higher content of pyridinic N. Leaching out of Co oxide from the matrix of CoO*_x_*/Co@GC‐NC led to lower ORR activity which indicates the synergy between cobalt and cobalt oxide components.

This work represents a significant progress in ORR catalyst attained by using non‐precious metal (metallic cobalt and cobalt oxide) and nitrogen‐doped carbon. In just less than a decade, the performance gap between non‐precious catalysts and Pt‐based catalysts have been tightened. Further work should ideally focus on its characterization in the membrane electrode assembly of PEMFC.

## Supporting information

As a service to our authors and readers, this journal provides supporting information supplied by the authors. Such materials are peer reviewed and may be re‐organized for online delivery, but are not copy‐edited or typeset. Technical support issues arising from supporting information (other than missing files) should be addressed to the authors.

Supplementary
