# Supplementary material for: Cobalt Oxide and Cobalt‐Graphitic Carbon Core–Shell Based Catalysts with Remarkably High Oxygen Reduction Reaction Activity
Source: Adv Sci (Weinh). 2016 Apr 23;3(9):1600060. doi: 10.1002/advs.201600060 (PMC5039978; doi:10.1002/advs.201600060)
Supplement: Supplementary file 1 — Supplementary [file ADVS-3-0j-s001.pdf]

Copyright WILEY-VCH Verlag GmbH & Co. KGaA, 69469 Weinheim, Germany, 2016.

ADVANCED SCIENCE
Open Access

Supporting Information

for *Adv. Sci.,* DOI: 10.1002/advs.201600060

Cobalt Oxide and Cobalt-Graphitic Carbon Core–Shell Based Catalysts with Remarkably High Oxygen Reduction Reaction Activity

Jie Yu, Gao Chen, Jaka Sunarso, Yinlong Zhu, Ran Ran, Zhonghua Zhu, Wei Zhou, and Zongping Shao**

Supporting Information

Cobalt oxide and cobalt-graphitic carbon core-shell based catalysts with remarkably high oxygen reduction reaction activity

Jie Yu, Gao Chen, Jaka Sunarso, Yinlong Zhu, Ran Ran, Zhonghua Zhu, Wei Zhou,* Zongping Shao*

Experimental Section

Catalysts synthesis

Synthesis of CoO_x/Co@GC-NC

The CoOx/Co@GC-NC was first prepared according to a procedure reported elsewhere [1]. Typically, a mixture of melamine (40g) and D(+)-glucosamine hydrochloride (1 g) were stirred thoroughly in 350ml water to form homogeneous solution. Cobalt(II) nitrate hexahydrate ($Co(NO_3)\cdot 6H_2O$) (0.95 g) and ethylenediaminetetraacetic acid (EDTA – 0.48 g) were mixed into 50ml water with the addition of 1-3ml ammonia solution(28 % in NH_3 in H_2O) dropwise to form ahomogeneous transparent solution. The two solution mixtures are mixed into one final mixture and stirred for several hours before heated overnight at 60 ℃ in an oil bath to form a roseate solid mixture. Following the grinding of solid into powder, the powder was first calcined at 600 ℃ for 1 hourfollowed by another calcinationat 800 ℃ for 1 hour. The calcination was performed in N_2 atmosphere. The ramp rate was 2.5℃ min^{-1}.The sample was cooled down to ambient temperature using 2.5 ^oC min^{-1} cooling rate.

Synthesis of CoO_x/Co@GC-NC-0 and CoO_x/Co@GC-NC-1

The CoO_x/Co@GC-NC-0 and CoO_x/Co@GC-NC-1 were obtained *via*identicalprocedure to CoO_x/Co@GC-NC, except that EDTA was not added in the synthesis of CoOx/Co@GC-NC-0 whilecitric acid monohydrate (CA) substituted EDTA in the synthesis of CoO_x/Co@GC-NC-1.

Synthesis of NC and NC-0

The NC and NC-0 were obtained *via* identical procedures to CoO_x/Co@GC-NC and CoO_x/Co@GC-NC-0, except with the absence of $CoNO_3.6H_2O$.

Synthesis of Co-EDTA and Co-CA

$CoNO_3.6H_2O$ (0.95g) and EDTA (40g) or CA (40g) were dissolved in 350ml water,followed by the addition of ~ 50ml ammonia solution. The following pyrolytic steps were identical to CoO_x/Co@GC-NC**.**

Synthesis of Co@GC-NC, Co@GC-NC-12MHCl-192h and CoO_x/Co@GC-NC-250℃-192h

The CoO_x/Co@GC-NC was etched intoCo@GC-NC in 3M HCl for 72hours at the room temperature to obtainCo@GC-NC. The Co@GC-NC was further etched with 12M HCl for 192hours at 60 ^{o}C to obtainCo@GC-NC-12MHCl-192h.The CoO_x/Co@GC-NC was subjected to oxidation by heating at 250 ^{o}C in air for 192hours to obtain CoO_x/Co@GC-NC-250℃-192h.

Characterizations

Powder X-ray diffraction (XRD, Rigaku Smartlab 3kW) was performed to identify the constituent phases of the synthesized catalysts using filtered Cu-Kα radiation (λ =1.5418Å) operating at a tube voltage of 40kV and a current of 200 mA. The diffraction patterns were obtained using a step scan at 2θ = 10-75 ° with a step size of 0.02 °.The morphology of the CoO_x/Co@GC-NC sample was obtained using a field emission scanning electron microscope (FE-SEM, HITACHI-S4800). The transmission electron microscopy (TEM) was conducted at 200 kV with FEI Tecnai G2T20 electron microscope and the corresponding EDX mappings were obtained using an FEI Tecnai G2F30 S-TWIN field emission transmission electron microscope equipped with EDAX operated at 300 kV. The chemical compositions and surface element states were probed by X-ray photoelectron spectroscopy (XPS, PHI5000 VersaProbe spectrometer equipped with an Al-Kα X-ray source). The Raman spectra were collected using an HR800 UV Raman microspectrometer (JOBIN YVON, France) with the green line of an argon laser as the excitation source. The specific surface area and pore size distribution were measured using N_2-adsorption on a BELSOR-MAX instrument.The elemental analysis results were acquired using a Vario EL elemental analyzer.

Electrodes preparation and electrochemical characterizations

For accurate comparison, analogous mass loading of catalyst was used in all electrochemical measurements performed on a CHI 760E Bipotentiostat. Electrochemical characterizations were conducted in a standard thee-electrode electrochemical cell with glassy carbon as the working electrode substrate, Pt wire as the counter electrode and Ag|AgCl (3.5 M KCl) as the reference electrode. Working electrode ink was prepared by dispersing 20 mg of each catalyst sample into a mixture of 100 μL of 5 wt % Nafion solution and 1 mL of ethanol followed by sonication for at least 1 hour. A 5 μL of ink aliquot was drop-casted onto glassy carbon substrate (5 mm diameter, 0.196 cm^2 area) and dried at room temperature inside an upside-down glass jar, leading to an approximate catalyst loading of 0.464 mg cm^{-2}.The commercial 20wt. % Pt/C catalyst (Johnson Matthey Corp.) was used as a comparison. Except for Pt/C case which used a quarter loading only of 5 mg, the working electrodes were prepared following the identical process which led to an approximate catalyst loading of 0.116 mg_{total} cm^{-2}.

For approximately 30 min prior to the start of each test till the end of test, O_2 or N_2 was continuously bubbled through 0.1 M KOH aqueous electrolyte solution. Cyclic

voltammetry (CV) and linear sweep voltammetry (LSV) were carried out using a rotating disk electrode (RDE). LSVs were performed at different rotation speeds (400, 800, 1200, 1600, 2000 and 2500 rpm) at a scan rate of 5 mV s^{-1} from 0.2 V to -0.6 V versus Ag|AgCl (3.5 M KCl) in O_2-saturated 0.1M KOH.Notably, the LSVs of the commercial 20 wt % Pt/C catalyst were recorded in the positive scan (−0.6 to 0.2 V) to avoid performance loss caused by anion adsorption. For CV, the scan rate was kept at 10 mV s^{-1} with potential from 0.1 V to -0.9 V versus Ag|AgCl (3.5 M KCl) in a N_2 or O_2 atmosphere.

Ag|AgCl (3.5 M KCl) was used as the reference electrode in all electrochemical measurements, which was calibrated to reversible hydrogenelectrode (RHE). The calibration was performed in the high purity hydrogen saturated0.1 M KOH solution with a platinum rotating disk electrode (0.126cm^2, Pine Research Instrumentation) as the working electrode. CV was cycled at a scan rate of 1$mVs^{-1}$from -0.8V to -1.15 V and the cross-over point at which the hydrogen evolution current changes into the hydrogen oxidation currents was taken as the thermodynamic (zero) potential for the hydrogen electrode reactions. In 0.1 M KOH, the zero current point lies at -0.950 V versus Ag|AgCl (3.5 M KCl); resulting in the correlation:

$$E\ (RHE) = E\ (Ag|AgCl\ (3.5\ M\ KCl)) + 0.950\ V \qquad (1)$$

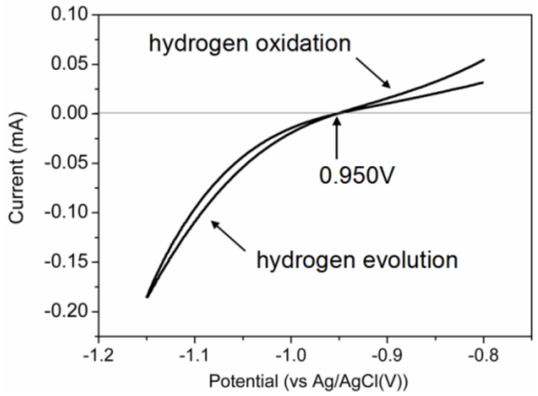

Figure S1. Potential calibration of the reference electrode in 0.1 M KOH solution.

The kinetic parameters such as the electron transfer number were determined using Koutecky-Levich (K-L) equation:

$$\frac{1}{J} = \frac{1}{J_K} + \frac{1}{J_L} = \frac{1}{nFkC^0} + \frac{1}{0.62nFD_{O_2}^{2/3}v^{-1/6}C^0\omega^{1/2}} \qquad (2)$$

Where J is the measured current density, J_L is the diffusion-limited current density, J_k is the mass-transport-corrected kinetic ORR current density, n is the electron transfer number, F is the Faraday constant, C^0 is the saturated concentration of oxygen in 0.1 M KOH, ω is the rotating rate (rad s^{-1}), D_{O2} is the diffusion coefficient of oxygen, v is the kinetic viscosity of the solution and k is the rate constant of ORR.

Rotating ring-disk electrode (RRDE) measurements were performed using Pt ring electrode to obtain the electron transfer number (*n*) and peroxide yield (*X*, percentage of HO_2^- relative to total products). For each measurement, the working (disk) electrode was scanned cathodically at 1600 rpm using a scan rate of 5 mV s^{-1} from 0.2 V to -0.6 V whereas the ring potential was set at 0.5 V versus Ag|AgCl (3.5 M KCl) to induce complete peroxide (OH_2^-) decomposition. The electron transfer number (*n*) and peroxide yield (*X*, % HO_2^-) were calculated as follows:

$$n = 4 \times \frac{I_d}{I_d + I_r / N} \quad (3)$$

$$\%HO_2^- = 200 \times \frac{I_r / N}{I_d + I_r / N} \quad (4)$$

Where I_d is the disk current, I_r is the ring current and *N* is the current collection efficiency of the Pt ring (0.422 according to earlier measurements).

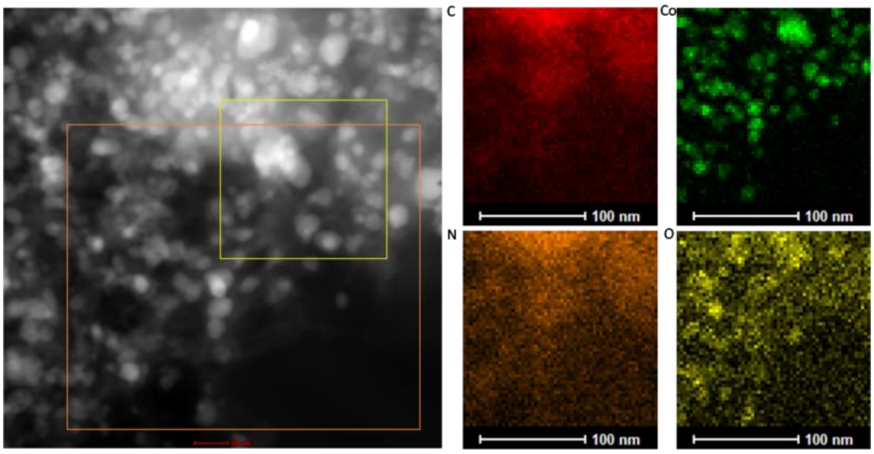

Figure S2. Transmission electron microscopy-energy dispersive X-ray elemental mapping of CoO_x/Co@GC-NC.

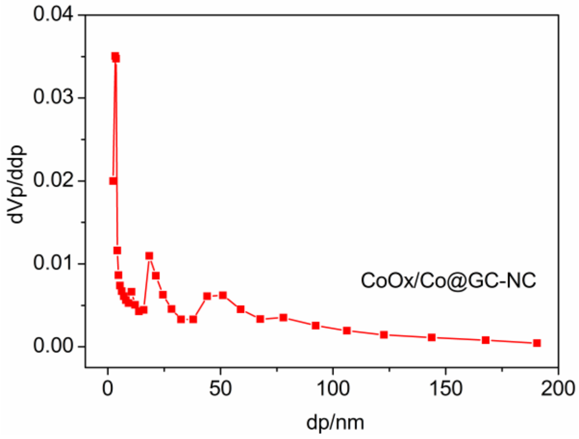

Figure S3. The respective Barrett-Joyner-Halenda pore size distribution profile of CoO_x/Co@GC-NC

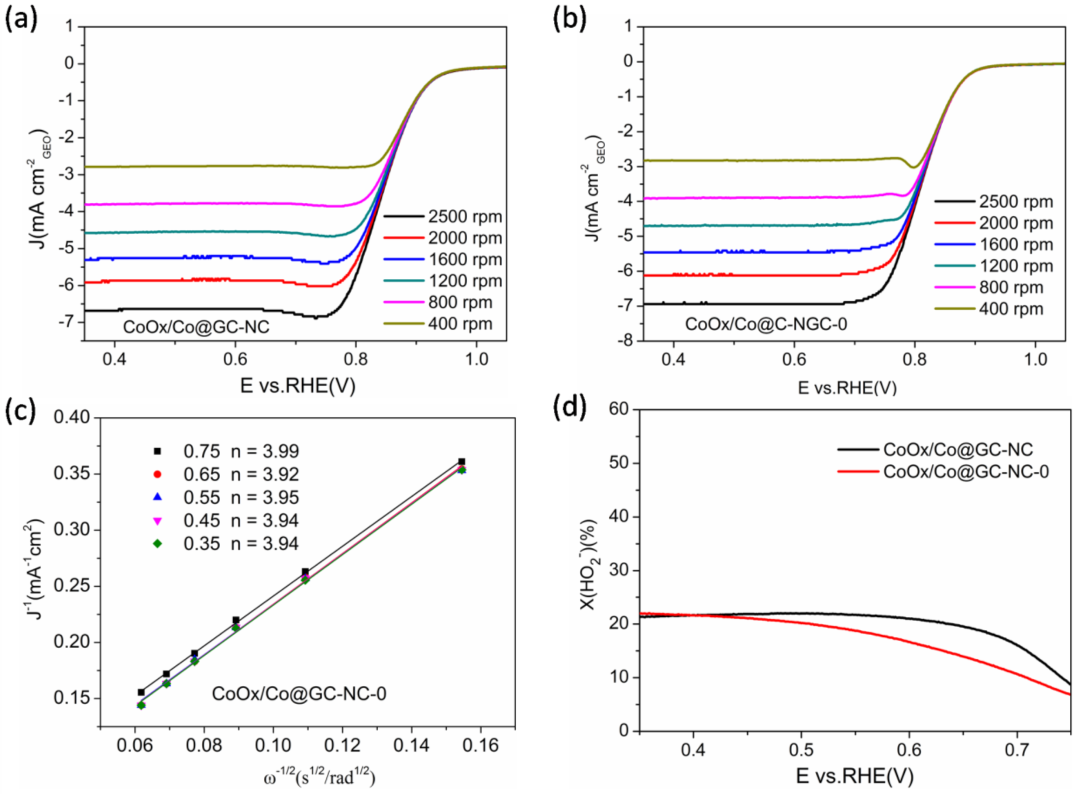

Figure S4. (a) Linear sweep voltammetry profilesof CoO$_x$/Co@GC-NC obtained using rotating disk electrode at 1600 rpm at different rotation rate in an O_2-saturated 0.1 M KOH solution at a scan rate of 5 mV s^{-1}; (b) Linear sweep voltammetry profiles of CoO$_x$/Co@GC-NC-0 obtained using rotating disk electrode at 1600 rpm at different rotation rate in an O_2-saturated 0.1 M KOH solution at a scan rate of 5 mV s^{-1}; c) The respective Koutecky-Levich plots at different potentials for CoO$_x$/Co@GC-NC-0 derived from Figure S4(b); d) Peroxide yield (X, percentage of HO_2^- relative to total products) at different potential from rotating ring disk electrode measurements.

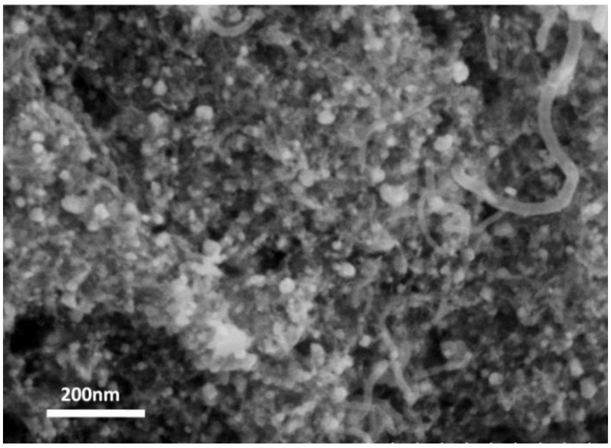

Figure S5.Scanning electron microscope image of CoO$_x$/Co@GC-NC-0.

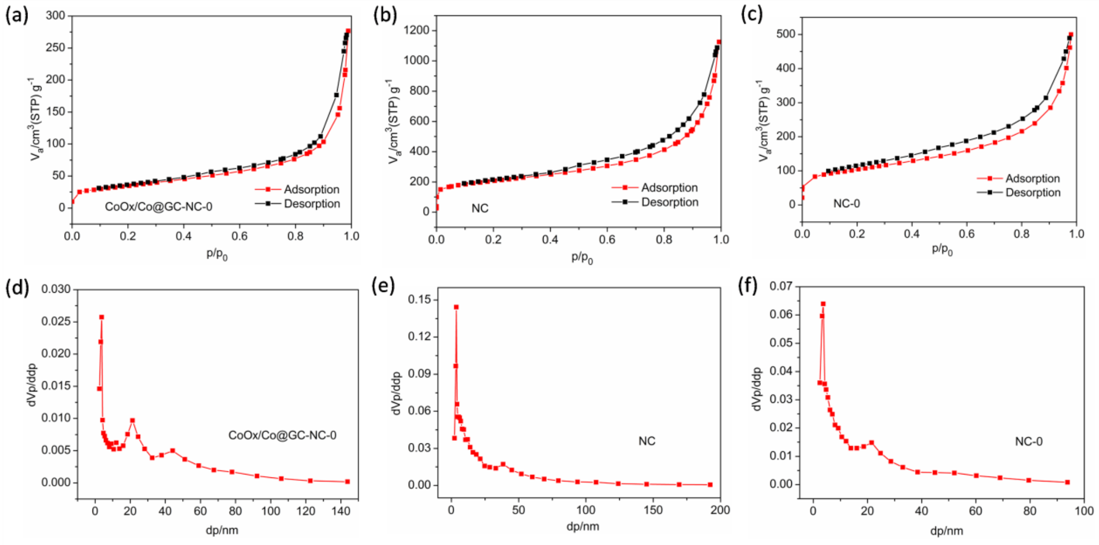

Figure S6. N_2 adsorption-desorption isotherms of (a) CoO_x/Co@GC-NC-0; (b) NC; and (c) NC-0; The respective Barrett-Joyner-Halenda pore size distribution profile of (d) CoO_x/Co@GC-NC-0; (e) NC; and (f) NC-0.

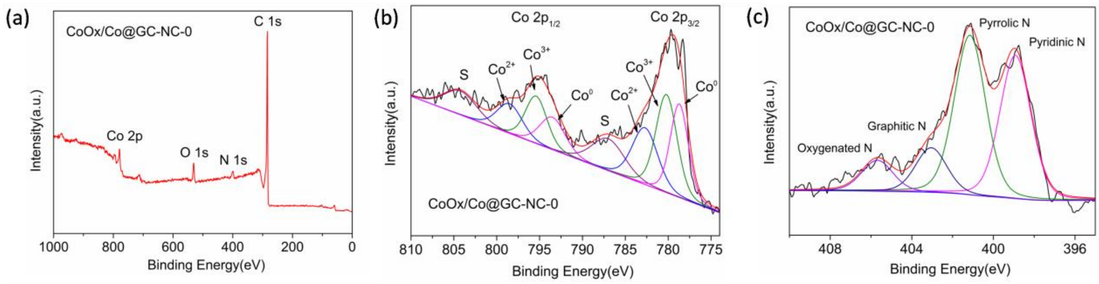

Figure S7. (a) X-ray photoelectron spectroscopy wide-scan spectrum of CoO_x/Co@GC-NC; (b, c) High-resolution N 1s and Co 2p XPS spectra of CoO_x/Co@GC-NC-0.

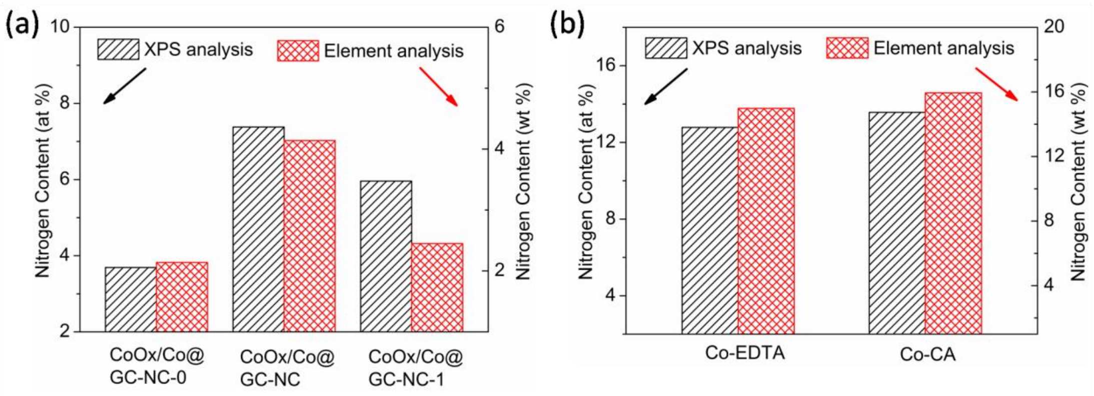

Figure S8. (a, b) Nitrogen content of CoO_x/Co@GC-NC-0, CoO_x/Co@GC-NC, CoO_x/Co@GC-NC-1, Co-EDTA and Co-CAobtained by elemental analysis (EA) and X-ray photoelectron spectra analysis.

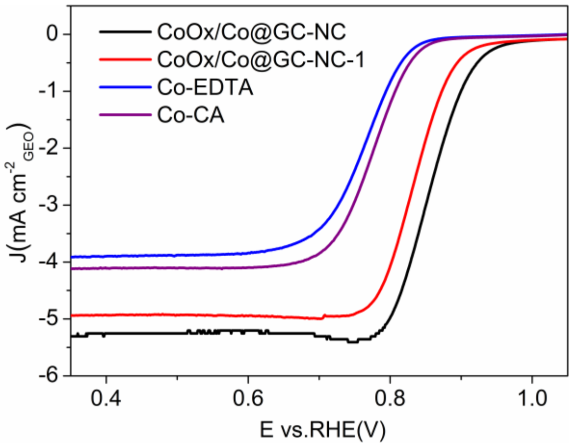

Figure S9. Linear sweep voltammetryprofiles of CoO_x/Co@GC-NC, CoO_x/Co@GC-NC-1, Co-EDTA and Co-CAobtained using rotating disk electrode at 1600 rpm in an O_2-saturated 0.1 M KOH solution at a scan rate of 5 mV s^{-1};

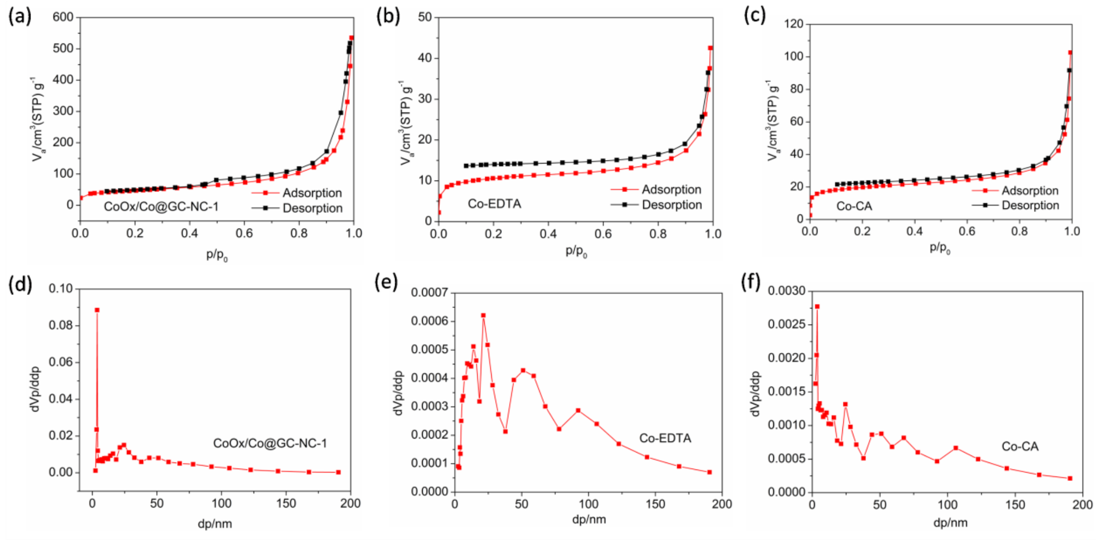

Figure S10. N_2 adsorption-desorption isotherms of (a) CoO_x/Co@GC-NC-1; (b) Co-EDTA; and (c) Co-CA; The respective Barrett-Joyner-Halenda pore size distribution profile of (d) CoO_x/Co@GC-NC-1; (e) Co-EDTA; and (f) Co-CA.

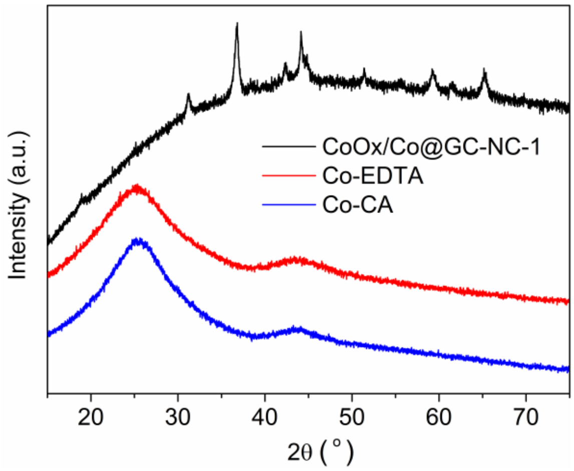

Figure S11. Powder X-ray diffraction patterns of CoO_x/Co@GC-NC-1, Co-EDTA and Co-CA.

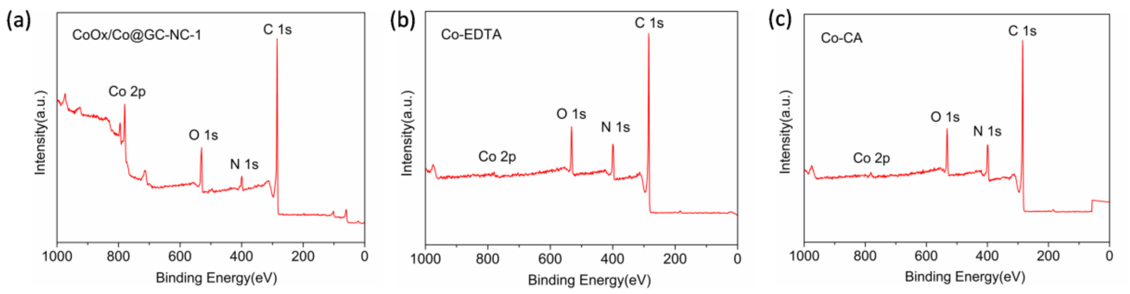

Figure S12. X-ray photoelectron spectroscopy wide-scan spectra of (a) CoO_x/Co@GC-NC-1; (b) Co-EDTA; and (c) Co-CA.

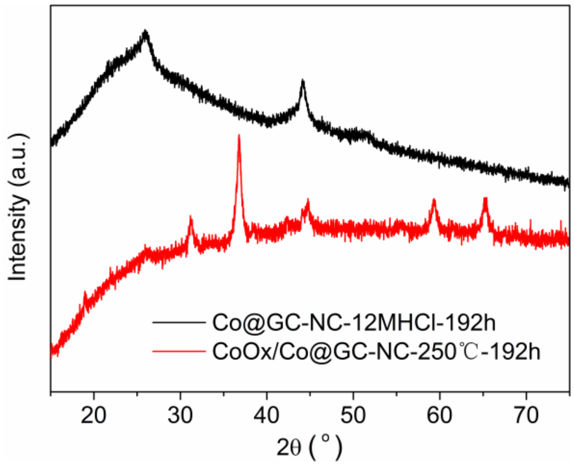

Figure S13. Powder X-ray diffraction patterns of Co@GC-NC-12MHCl-192h and CoO_x/Co@GC-NC-250℃-192h.

Table S1. Summary of the pore structure parameters of the different samples.

Sample	S_{BET}[a] (m^2g^{-1})	V[b] (cm^3g^{-1})
CoO_x/Co@GC-NC	148.03	0.6052
CoO_x/Co@GC-NC-0	119.9	0.4148
NC	685.15	1.5883
NC-0	350.74	0.7103
CoO_x/Co@GC-NC-1	153.74	0.8030
Co-EDTA	34.58	0.0461
Co-CA	64.74	0.1152

[a]S_{BET}: specific surface area from BET method. [b]V: pore volume

Table S2. Relative atomic content of different N speciesfor CoO_x/Co@GC-NC, CoO_x/Co@GC-NC-0, CoO_x/Co@GC-NC-1, Co-EDTA and Co-CA samples from their N1s spectra deconvolution.

Sample	Pyridinic N	Pyrrolic N	Graphitic N	Oxygenated N
CoO_x/Co@GC-NC	0.545	0.335	0.077	0.044
CoO_x/Co@GC-NC-0	0.379	0.422	0.118	0.082
CoO_x/Co@GC-NC-1	0.400	0.431	0.097	0.072
Co-EDTA	0.438	0.443	0.079	0.039
Co-CA	0.410	0.506	0.065	0.019

Table S3. Comparison of the electrocatalytic activity of CoO_x/Co@GC-NC to other recently reported catalysts for ORR in an alkaline solution.

Catalyst	Loading($mgcm^{-2}$)	E_{onset}(V vs. RHE)	$E_{1/2}$(V vs. RHE)	Reference
CoO_x/Co@GC-NC	0.464	0.974	0.858	*This work*
Fe-CNT-PA	0.5	---	~-0.18 vs Ag|AgCl	*Energy Environ. Sci. 2015, 8, 1799.*[2]
Co@Co_3O_4@C-CM	0.1	0.93	0.81	*Energy Environ. Sci. 2015, 8, 568.*[3]
Co_3O_4/N-rmGO	0.1	---	0.83	*Nat. Mater. 2011, 10, 780.*[4]
Fe-N-CNFs	0.6	-0.02 vs Ag|AgCl	-0.14 vs Ag|AgCl	*Angew. Chem. Int. Ed. 2015, 54, 8179.*[5]
Co_3O_4/CNW	0.128	~-0.15 vs SCE	---	J. Mater. Chem. A, 2015, 3, 11615.[6]
Co/N-CNTs	~0.2	0.94	0.84	J. Mater. Chem. A, 2016, (DOI:10.1039/C5TA10551J).[7]
N-doped Fe/Fe_3C@C/RGO	0.707	1	0.93	*Adv. Energy. Mater, 2014, 4, 1400337.*[8]
$Co_{1-x}S$/RGO	0.1	~0.87	---	*Angew. Chem. Int. Ed. 2011, 50, 10969.*[9]
PCN-FeCo/C	0.2	~1.0	0.85	*Adv. Mater. 2015, 27, 3431.*[10]
MnO-m-N-C	0.1	---	0.81	*Adv. Funct. Mater, 2012, 22, 4584.*[11]
Fe_3C/C-800	0.6	1.05	0.83	*Angew. Chem. Int. Ed. 2014, 53, 3675.*[12]
Fe-N-C/G	0.23	---	~0.84	*J. Mater. Chem. A, 2015,DOI:10.1039/C5TA09232A.*[13]
G-Co/CoO	---	---	-0.176 vs Ag|AgCl	*Angew.Chem.Int.Ed. 2012, 51, 11770.*[14]
Co_3O_4 OC/RGO	0.4	---	-0.14V vs Hg/HgO	*Sci. Rep. 2013, 3, 2300.*[15]

References

[1] a) J. Wang, Z. Xu, Y. T. Gong, C. L. Han, H. R. Li, Y. Wang, *ChemCatChem*, **2014**, *6*, 1204-1209; b) D. F. Su, J. Wang, H. Y.Jin, Y. T. Gong, M. M. Li, Z. F. Pang, Y. Wang, *J. Mater. Chem. A* **2015**, *3*, 11756-11761.

[2] G. Yang, W. Choi, X. Pu, C. Yu, *Energy Environ. Sci.***2015**, *8*, 1799-1807.

[3] W. Xia, R. Q. Zou, L. An, D. G. Xia, S. J. Guo, *Energy Environ. Sci.***2015**, *8*,568-576.

[4] Y. Y. Liang, Y. G. Li, H. L. Wang, J. G. Zhou, J. Wang, T. Regier, H. J. Dai, *Nat. Mater.* **2011**, *10*, 780-786.

[5] Z. Y. Wu, X. X. Xu, B. C. Hu, H. W. Liang, Y. Lin, L. F. Chen, S. H. Yu, *Angew. Chem. Int. Ed.***2015**, *54*, 8179-8183.

[6] S. Y. Liu, L. J. Li, H. S. Ahn, A. Manthiram, *J. Mater. Chem. A*, **2015**, *3*, 11615-11623.

[7] Y. Y. Liu, H. L. Jiang, Y. H. Zhu, X. L. Yang, C. Z. Li,*J. Mater. Chem. A*,**2016**, DOI: 10.1039/C5TA10551J.

[8] Y. Hou, T. Z. Huang, Z. H. Wen, S. Mao, S. M. Cui, J. H. Chen, *Adv. Energy Mater.***2014**, *4*, 1400337.

[9] H. L. Wang, Y. Y. Liang, Y. G. Li, H. J. Dai *Angew. Chem. Int. Ed.***2011**, *50*, 10969-10972.

[10] Q. P. Lin, X. H. Bu, A. G. Kong, C. Y. Mao, F. Bu, P. Y. Feng, *Adv. Mater.***2015**, *27*, 3431-3436.

[11] Y. M. Tan, C. F. Xu, G. X. Chen, X. L. Fang, N. F. Zheng, Q. J. Xie, *Adv. Funct. Mater.***2012**, *22*, 4584-4591.

[12] Y. Hu, J. O. Jensen, W. Zhang, L. N. Cleemann, W. Xing, N. J. Bjerrum, Q. F. Li, *Angew. Chem. Int. Ed.***2014**, *53*, 3675 -3679.

[13] D. P. He, Y. L. Xiong, J. L. Yang, X. Chen, Z. X. Deng, M. Pan, Y. D. Li, S. C. Mu, *J. Mater. Chem. A*, **2015**(DOI:10.1039/C5TA09232A).

[14] S. J. Guo, S. Zhang, L. H. Wu, S. H. Sun, *Angew. Chem. Int. Ed.***2012**, *51*, 11770-11773.

[15] J. W. Xiao, Q. Kuang, S. H. Yang, F. Xiao, S. Wang, L. Guo, *Sci. Rep.***2013**, *3*, 2300-2307.
